# Supplementary material for: Patient education for older adults with cancer and their caregivers: Protocol for a scoping review
Source: PLoS One. 2025 Jul 8;20(7):e0327383. doi: 10.1371/journal.pone.0327383 (PMC12237015; doi:10.1371/journal.pone.0327383)
Supplement: S2 File — (DOCX) [file pone.0327383.s002.docx]

**Supplemental File 1 Medline search**

**Ovid MEDLINE(R) ALL 2000 to January 23, 2025**

| **#** | **Searches** | **Results** | **Type** |
| --- | --- | --- | --- |
| 1 | exp Neoplasms/ | 4065294 | Advanced |
| 2 | neoplas*.tw,kf. | 476635 | Advanced |
| 3 | paraneoplas*.tw,kf. | 13964 | Advanced |
| 4 | cancer*.tw,kf. | 2500273 | Advanced |
| 5 | tumo?r*.tw,kf. | 2210727 | Advanced |
| 6 | onco*.tw,kf. | 461217 | Advanced |
| 7 | metast*.tw,kf. | 685529 | Advanced |
| 8 | multimetast*.tw,kf. | 55 | Advanced |
| 9 | macrometast*.tw,kf. | 1181 | Advanced |
| 10 | micrometast*.tw,kf. | 7510 | Advanced |
| 11 | malig*.tw,kf. | 751226 | Advanced |
| 12 | aberrant crypt foci.tw,kf. | 1633 | Advanced |
| 13 | acanthoma*.tw,kf. | 785 | Advanced |
| 14 | acrospiroma*.tw,kf. | 91 | Advanced |
| 15 | adamantinom*.tw,kf. | 1348 | Advanced |
| 16 | adenocarc*.tw,kf. | 198303 | Advanced |
| 17 | adenofibrom*.tw,kf. | 611 | Advanced |
| 18 | adenolymphom*.tw,kf. | 481 | Advanced |
| 19 | adenomat*.tw,kf. | 21262 | Advanced |
| 20 | adenomyo*.tw,kf. | 5686 | Advanced |
| 21 | adenosarcom*.tw,kf. | 706 | Advanced |
| 22 | adenosquam*.tw,kf. | 3429 | Advanced |
| 23 | ameloblastom*.tw,kf. | 4235 | Advanced |
| 24 | androblastom*.tw,kf. | 99 | Advanced |
| 25 | angiofibrom*.tw,kf. | 2565 | Advanced |
| 26 | angiokeratom*.tw,kf. | 1153 | Advanced |
| 27 | angiolipom*.tw,kf. | 723 | Advanced |
| 28 | angioma*.tw,kf. | 12324 | Advanced |
| 29 | angiomyolipom*.tw,kf. | 4845 | Advanced |
| 30 | angiomyom*.tw,kf. | 178 | Advanced |
| 31 | angiosarcom*.tw,kf. | 7985 | Advanced |
| 32 | apudoma*.tw,kf. | 302 | Advanced |
| 33 | arrhenoblastom*.tw,kf. | 355 | Advanced |
| 34 | astrocytom*.tw,kf. | 18868 | Advanced |
| 35 | blastom*.tw,kf. | 10659 | Advanced |
| 36 | Bowen*.tw,kf. | 3609 | Advanced |
| 37 | Brenner*.tw,kf. | 1710 | Advanced |
| 38 | Buschke-Lowenstein*.tw,kf. | 321 | Advanced |
| 39 | carcin*.tw,kf. | 1003667 | Advanced |
| 40 | cementoma*.tw,kf. | 225 | Advanced |
| 41 | chemodectomas*.tw,kf. | 314 | Advanced |
| 42 | cholangiocarcin*.tw,kf. | 20795 | Advanced |
| 43 | chondroblastom*.tw,kf. | 1236 | Advanced |
| 44 | chondroma*.tw,kf. | 4851 | Advanced |
| 45 | chordoma*.tw,kf. | 5084 | Advanced |
| 46 | chondrosarcom*.tw,kf. | 9690 | Advanced |
| 47 | choriocarcin*.tw,kf. | 7750 | Advanced |
| 48 | craniopharyngioma*.tw,kf. | 5461 | Advanced |
| 49 | cystadenofibrom*.tw,kf. | 242 | Advanced |
| 50 | cystosarcom*.tw,kf. | 653 | Advanced |
| 51 | cytoma*.tw,kf. | 405 | Advanced |
| 52 | dermatofibrosarcom*.tw,kf. | 2471 | Advanced |
| 53 | desmoplas*.tw,kf. | 6280 | Advanced |
| 54 | dysgerminoma*.tw,kf. | 1551 | Advanced |
| 55 | DCIS.tw,kf. | 6324 | Advanced |
| 56 | DSRCT.tw,kf. | 453 | Advanced |
| 57 | ependymom*.tw,kf. | 6526 | Advanced |
| 58 | Ewing*.tw,kf. | 12174 | Advanced |
| 59 | fibroadenom*.tw,kf. | 4457 | Advanced |
| 60 | fibroepithelial*.tw,kf. | 1408 | Advanced |
| 61 | fibroma*.tw,kf. | 15000 | Advanced |
| 62 | fibrosarcom*.tw,kf. | 12872 | Advanced |
| 63 | FAMMM.tw,kf. | 89 | Advanced |
| 64 | gangliogliom*.tw,kf. | 1685 | Advanced |
| 65 | ganglioneurom*.tw,kf. | 2542 | Advanced |
| 66 | gastrinoma*.tw,kf. | 1840 | Advanced |
| 67 | germinoma*.tw,kf. | 2239 | Advanced |
| 68 | glioblastom*.tw,kf. | 56249 | Advanced |
| 69 | glioma*.tw,kf. | 78566 | Advanced |
| 70 | gliosarcom*.tw,kf. | 1291 | Advanced |
| 71 | glomus jugulare*.tw,kf. | 909 | Advanced |
| 72 | glomus tympanicum*.tw,kf. | 240 | Advanced |
| 73 | glucagonoma*.tw,kf. | 1017 | Advanced |
| 74 | gonadoblastom*.tw,kf. | 913 | Advanced |
| 75 | GCTOB.tw,kf. | 11 | Advanced |
| 76 | GIST?.tw,kf. | 10507 | Advanced |
| 77 | h?emangioendotheliom*.tw,kf. | 4101 | Advanced |
| 78 | h?emangiom*.tw,kf. | 27557 | Advanced |
| 79 | h?emangiopericytom*.tw,kf. | 3758 | Advanced |
| 80 | h?emangiosarcom*.tw,kf. | 1552 | Advanced |
| 81 | hamartoblastom*.tw,kf. | 49 | Advanced |
| 82 | hepatoblastom*.tw,kf. | 4418 | Advanced |
| 83 | hepatoma*.tw,kf. | 31110 | Advanced |
| 84 | histiocytom*.tw,kf. | 6048 | Advanced |
| 85 | hodgkin*.tw,kf. | 77178 | Advanced |
| 86 | nonhodgkin*.tw,kf. | 139 | Advanced |
| 87 | (hutchinson* adj2 freckle*).tw,kf. | 63 | Advanced |
| 88 | HNPCC.tw,kf. | 2340 | Advanced |
| 89 | immunocytom*.tw,kf. | 622 | Advanced |
| 90 | incidentaloma?.tw,kf. | 3062 | Advanced |
| 91 | insulinoma*.tw,kf. | 7465 | Advanced |
| 92 | kasabach merrit*.tw,kf. | 892 | Advanced |
| 93 | leiomyoblastom*.tw,kf. | 404 | Advanced |
| 94 | leiomyom*.tw,kf. | 18172 | Advanced |
| 95 | leiomyosarcom*.tw,kf. | 12243 | Advanced |
| 96 | leuk?em*.tw,kf. | 319890 | Advanced |
| 97 | preleuk?em*.tw,kf. | 1860 | Advanced |
| 98 | leukoplak*.tw,kf. | 5781 | Advanced |
| 99 | li-fraumeni*.tw,kf. | 1718 | Advanced |
| 100 | lipoblastom*.tw,kf. | 598 | Advanced |
| 101 | lipoma*.tw,kf. | 17578 | Advanced |
| 102 | liposarcom*.tw,kf. | 8385 | Advanced |
| 103 | luteoma*.tw,kf. | 259 | Advanced |
| 104 | lymphangio*.tw,kf. | 16122 | Advanced |
| 105 | lymphoblastom*.tw,kf. | 375 | Advanced |
| 106 | lymphocytom*.tw,kf. | 378 | Advanced |
| 107 | lymphoma*.tw,kf. | 221541 | Advanced |
| 108 | lymphosarcom*.tw,kf. | 5252 | Advanced |
| 109 | lynch*.tw,kf. | 6261 | Advanced |
| 110 | macroglobulinem*.tw,kf. | 4469 | Advanced |
| 111 | m?croprolactinom*.tw,kf. | 983 | Advanced |
| 112 | mastocytom*.tw,kf. | 1929 | Advanced |
| 113 | mastocytos?s*.tw,kf. | 4643 | Advanced |
| 114 | medulloblastom*.tw,kf. | 10295 | Advanced |
| 115 | meigs*.tw,kf. | 969 | Advanced |
| 116 | melanoameloblastom*.tw,kf. | 10 | Advanced |
| 117 | melanoblastom*.tw,kf. | 476 | Advanced |
| 118 | melanocarcin*.tw,kf. | 105 | Advanced |
| 119 | melanoma*.tw,kf. | 150275 | Advanced |
| 120 | melanosis.tw,kf. | 2835 | Advanced |
| 121 | melanotic*.tw,kf. | 2868 | Advanced |
| 122 | meningiom*.tw,kf. | 26612 | Advanced |
| 123 | mesenchymom*.tw,kf. | 871 | Advanced |
| 124 | mesoblast*.tw,kf. | 813 | Advanced |
| 125 | mesonephrom*.tw,kf. | 123 | Advanced |
| 126 | mesotheliom*.tw,kf. | 19750 | Advanced |
| 127 | metaplas*.tw,kf. | 26730 | Advanced |
| 128 | muir-torre*.tw,kf. | 572 | Advanced |
| 129 | myelolipom*.tw,kf. | 1289 | Advanced |
| 130 | myoepitheliom*.tw,kf. | 879 | Advanced |
| 131 | myofibrom*.tw,kf. | 817 | Advanced |
| 132 | myeloma*.tw,kf. | 68332 | Advanced |
| 133 | myoma*.tw,kf. | 7207 | Advanced |
| 134 | myosarcom*.tw,kf. | 239 | Advanced |
| 135 | myxofibrosarcom*.tw,kf. | 846 | Advanced |
| 136 | myxoma*.tw,kf. | 10849 | Advanced |
| 137 | myxosarcom*.tw,kf. | 288 | Advanced |
| 138 | n?evocarcin*.tw,kf. | 78 | Advanced |
| 139 | neurilemmom*.tw,kf. | 2457 | Advanced |
| 140 | neurocytom*.tw,kf. | 893 | Advanced |
| 141 | neuroectodermal*.tw,kf. | 7539 | Advanced |
| 142 | neurofibroma*.tw,kf. | 21711 | Advanced |
| 143 | neurofibrosarcom*.tw,kf. | 431 | Advanced |
| 144 | neurilemmom*.tw,kf. | 2457 | Advanced |
| 145 | neuroblastom*.tw,kf. | 43384 | Advanced |
| 146 | neuroma*.tw,kf. | 11831 | Advanced |
| 147 | neurothekeom*.tw,kf. | 289 | Advanced |
| 148 | NSCLC.tw,kf. | 69087 | Advanced |
| 149 | odontoma*.tw,kf. | 1705 | Advanced |
| 150 | oligo*.tw,kf. | 318051 | Advanced |
| 151 | osteoblastom*.tw,kf. | 1357 | Advanced |
| 152 | osteochondrom*.tw,kf. | 3772 | Advanced |
| 153 | osteoclastom*.tw,kf. | 398 | Advanced |
| 154 | osteoma*.tw,kf. | 12678 | Advanced |
| 155 | osteosarcom*.tw,kf. | 31522 | Advanced |
| 156 | papilloma*.tw,kf. | 75295 | Advanced |
| 157 | papillary*.tw,kf. | 70678 | Advanced |
| 158 | paragangliom*.tw,kf. | 9610 | Advanced |
| 159 | pheochromocytom*.tw,kf. | 20410 | Advanced |
| 160 | phyllo?des*.tw,kf. | 2849 | Advanced |
| 161 | pinealocytoma*.tw,kf. | 18 | Advanced |
| 162 | pinealoma*.tw,kf. | 366 | Advanced |
| 163 | pineoblastoma*.tw,kf. | 536 | Advanced |
| 164 | pineocytoma*.tw,kf. | 297 | Advanced |
| 165 | plasmacytom*.tw,kf. | 7586 | Advanced |
| 166 | (polycythem* adj2 vera?).tw,kf. | 6749 | Advanced |
| 167 | prolactinom*.tw,kf. | 4048 | Advanced |
| 168 | retinoblastom*.tw,kf. | 18862 | Advanced |
| 169 | rhabdoid*.tw,kf. | 3656 | Advanced |
| 170 | rhabdomyom*.tw,kf. | 1833 | Advanced |
| 171 | rhabdomyosarcom*.tw,kf. | 14245 | Advanced |
| 172 | sarcom*.tw,kf. | 133063 | Advanced |
| 173 | seminoma*.tw,kf. | 8348 | Advanced |
| 174 | Sertoli- Leydig.tw,kf. | 796 | Advanced |
| 175 | somatostatinoma*.tw,kf. | 408 | Advanced |
| 176 | somatotrophinom*.tw,kf. | 90 | Advanced |
| 177 | struma ovarii*.tw,kf. | 719 | Advanced |
| 178 | thecoma*.tw,kf. | 501 | Advanced |
| 179 | teratocarcin*.tw,kf. | 2800 | Advanced |
| 180 | teratoma*.tw,kf. | 17873 | Advanced |
| 181 | thymom*.tw,kf. | 11327 | Advanced |
| 182 | trophoblast*.tw,kf. | 27560 | Advanced |
| 183 | vipoma*.tw,kf. | 434 | Advanced |
| 184 | wilms*.tw,kf. | 11570 | Advanced |
| 185 | or/1-184 | 6134582 | Advanced |
| 186 | Patients/ | 25713 | Advanced |
| 187 | Outpatients/ | 23328 | Advanced |
| 188 | Caregivers/ | 56008 | Advanced |
| 189 | exp Family/ | 386256 | Advanced |
| 190 | (patient? or outpatient?).tw,kf. | 8894168 | Advanced |
| 191 | (caregiv* or care-giv* or carer?).tw,kf. | 137185 | Advanced |
| 192 | (family or families).tw,kf. | 1246378 | Advanced |
| 193 | or/186-192 | 10085925 | Advanced |
| 194 | exp Education, Special/ | 15783 | Advanced |
| 195 | exp Teaching/ | 96385 | Advanced |
| 196 | exp Teaching Materials/ | 125811 | Advanced |
| 197 | Multimedia/ | 2300 | Advanced |
| 198 | Internet-Based Intervention/ | 1557 | Advanced |
| 199 | exp Audiovisual Aids/ | 115231 | Advanced |
| 200 | exp Videoconferencing/ | 2990 | Advanced |
| 201 | exp Video-Audio Media/ | 44589 | Advanced |
| 202 | Webcast/ | 1161 | Advanced |
| 203 | Webcasts as Topic/ | 475 | Advanced |
| 204 | Postcards as Topic/ | 8 | Advanced |
| 205 | exp Tape Recording/ | 15940 | Advanced |
| 206 | Videotape Recording/ | 11693 | Advanced |
| 207 | Pamphlets/ | 4205 | Advanced |
| 208 | or/194-207 | 280157 | Advanced |
| 209 | 193 and 208 | 65529 | Advanced |
| 210 | Patient Education as Topic/ | 89934 | Advanced |
| 211 | Consumer Health Information/ | 4522 | Advanced |
| 212 | Health Communication/ | 3566 | Advanced |
| 213 | Patient Education Handout/ | 5867 | Advanced |
| 214 | ((educat* or psychoed*) adj3 (patient? or outpatient?)).tw,kf. | 57002 | Advanced |
| 215 | ((educat* or psychoed*) adj3 (caregiv* or care-giv* or carer? or family or families)).tw,kf. | 18704 | Advanced |
| 216 | (teach* adj3 (patient? or outpatient?)).tw,kf. | 7177 | Advanced |
| 217 | (teach* adj3 (caregiv* or care-giv* or carer? or family or families)).tw,kf. | 3050 | Advanced |
| 218 | (educat* adj5 communicat* adj3 (patient? or outpatient?)).tw,kf. | 1206 | Advanced |
| 219 | (educat* adj5 communicat* adj3 (caregiv* or care-giv* or carer? or family or families)).tw,kf. | 287 | Advanced |
| 220 | (teach* adj5 communicat* adj3 (patient? or outpatient?)).tw,kf. | 252 | Advanced |
| 221 | (teach* adj5 communicat* adj3 (caregiv* or care-giv* or carer? or family or families)).tw,kf. | 66 | Advanced |
| 222 | (educat* adj5 material? adj3 (patient? or outpatient?)).tw,kf. | 2400 | Advanced |
| 223 | (educat* adj5 material? adj3 (caregiv* or care-giv* or carer? or family or families)).tw,kf. | 152 | Advanced |
| 224 | (teach* adj5 material? adj3 (patient? or outpatient?)).tw,kf. | 55 | Advanced |
| 225 | (teach* adj5 material? adj3 (caregiv* or care-giv* or carer? or family or families)).tw,kf. | 28 | Advanced |
| 226 | (educat* adj5 program* adj3 (patient? or outpatient?)).tw,kf. | 3777 | Advanced |
| 227 | (educat* adj5 program* adj3 (caregiv* or care-giv* or carer? or family or families)).tw,kf. | 1258 | Advanced |
| 228 | (teach* adj5 program* adj3 (patient? or outpatient?)).tw,kf. | 281 | Advanced |
| 229 | (teach* adj5 program* adj3 (caregiv* or care-giv* or carer? or family or families)).tw,kf. | 172 | Advanced |
| 230 | (educat* adj5 (multimedia* or multi-media*) adj3 (patient? or outpatient?)).tw,kf. | 110 | Advanced |
| 231 | (educat* adj5 (multimedia* or multi-media*) adj3 (caregiv* or care-giv* or carer? or family or families)).tw,kf. | 7 | Advanced |
| 232 | (teach* adj5 (multimedia* or multi-media*) adj3 (patient? or outpatient?)).tw,kf. | 4 | Advanced |
| 233 | (teach* adj5 (multimedia* or multi-media*) adj3 (caregiv* or care-giv* or carer? or family or families)).tw,kf. | 2 | Advanced |
| 234 | (educat* adj5 (audiovisual* or audio-visual*) adj3 (patient? or outpatient?)).tw,kf. | 105 | Advanced |
| 235 | (educat* adj5 (audiovisual* or audio-visual*) adj3 (caregiv* or care-giv* or carer? or family or families)).tw,kf. | 6 | Advanced |
| 236 | (teach* adj5 (audiovisual* or audio-visual*) adj3 (patient? or outpatient?)).tw,kf. | 2 | Advanced |
| 237 | (teach* adj5 (audiovisual* or audio-visual*) adj3 (caregiv* or care-giv* or carer? or family or families)).tw,kf. | 4 | Advanced |
| 238 | (educat* adj5 video* adj3 (patient? or outpatient?)).tw,kf. | 599 | Advanced |
| 239 | (educat* adj5 video* adj3 (caregiv* or care-giv* or carer? or family or families)).tw,kf. | 41 | Advanced |
| 240 | (educat* adj5 tape* adj3 (patient? or outpatient?)).tw,kf. | 15 | Advanced |
| 241 | (educat* adj5 tape* adj3 (caregiv* or care-giv* or carer? or family or families)).tw,kf. | 1 | Advanced |
| 242 | (educat* adj5 (class or classroom?) adj3 (patient? or outpatient?)).tw,kf. | 76 | Advanced |
| 243 | (educat* adj5 (class or classroom?) adj3 (caregiv* or care-giv* or carer? or family or families)).tw,kf. | 100 | Advanced |
| 244 | (teach* adj5 (class or classroom?) adj3 (patient? or outpatient?)).tw,kf. | 20 | Advanced |
| 245 | (teach* adj5 (class or classroom?) adj3 (caregiv* or care-giv* or carer? or family or families)).tw,kf. | 16 | Advanced |
| 246 | (educat* adj5 information* adj3 (patient? or outpatient?)).tw,kf. | 1513 | Advanced |
| 247 | (educat* adj5 information* adj3 (caregiv* or care-giv* or carer? or family or families)).tw,kf. | 331 | Advanced |
| 248 | (teach* adj5 information* adj3 (patient? or outpatient?)).tw,kf. | 87 | Advanced |
| 249 | (teach* adj5 information* adj3 (caregiv* or care-giv* or carer? or family or families)).tw,kf. | 37 | Advanced |
| 250 | ((workshop? or work-shop?) adj3 (patient? or outpatient?)).tw,kf. | 624 | Advanced |
| 251 | ((workshop? or work-shop?) adj3 (caregiv* or care-giv* or carer? or family or families)).tw,kf. | 367 | Advanced |
| 252 | (seminar? adj3 (patient? or outpatient?)).tw,kf. | 161 | Advanced |
| 253 | (seminar? adj3 (caregiv* or care-giv* or carer? or family or families)).tw,kf. | 80 | Advanced |
| 254 | (session? adj3 (patient? or outpatient?)).tw,kf. | 8925 | Advanced |
| 255 | (session? adj3 (caregiv* or care-giv* or carer? or family or families)).tw,kf. | 1154 | Advanced |
| 256 | webinar?.tw,kf. | 2083 | Advanced |
| 257 | webcast?.tw,kf. | 299 | Advanced |
| 258 | podcast?.tw,kf. | 1759 | Advanced |
| 259 | pamphlet?.tw,kf. | 2551 | Advanced |
| 260 | booklet?.tw,kf. | 4690 | Advanced |
| 261 | brochure?.tw,kf. | 3036 | Advanced |
| 262 | (handout? or hand-out?).tw,kf. | 1808 | Advanced |
| 263 | or/209-262 | 248846 | Advanced |
| 264 | 185 and 263 | 32044 | Advanced |
| 265 | limit 264 to "humans only (removes records about animals)" | 31880 | Advanced |
| 266 | limit 265 to (clinical conference or clinical trial, veterinary or clinical trial protocol or news or newspaper article or preprint) | 263 | Advanced |
| 267 | 265 not 266 | 31617 | Advanced |
| 268 | limit 267 to elderly | 9342 | Advanced |
| 269 | limit 268 to yr="2000 -Current" | 8226 | Advanced |
|  | | |  |
