# Supplementary material for: Patient education for older adults with cancer and their caregivers: Protocol for a scoping review
Source: PLoS One. 2025 Jul 8;20(7):e0327383. doi: 10.1371/journal.pone.0327383 (PMC12237015; doi:10.1371/journal.pone.0327383)
Supplement: S3 File — (DOCX) [file pone.0327383.s003.docx]

**Table 1. Characteristics of Included Studies**

| **Category** | **Information to Extract** | **Study 1**  Author/ Year | **Study 2**  Author/ Year | **Study 3**  Author/ Year | **Study 4**  Author/ Year | **Study 5**  Author/ Year | ... |
| --- | --- | --- | --- | --- | --- | --- | --- |
| **Basic Study Information** |  |  |  |  |  |  |  |
|  | Country |  |  |  |  |  |  |
|  | Study Type |  |  |  |  |  |  |
|  | Funding Source |  |  |  |  |  |  |
| **Study Population** |  |  |  |  |  |  |  |
|  | Participant Characteristics (age, gender, cancer type, cancer stage, treatment options, etc.) |  |  |  |  |  |  |
|  | Caregiver Characteristics (Relationship to patient, age, gender, etc.) |  |  |  |  |  |  |
|  | Sample Size |  |  |  |  |  |  |
| **Patient Education** |  |  |  |  |  |  |  |
|  | Educational Content |  |  |  |  |  |  |
|  | Duration |  |  |  |  |  |  |
|  | Frequency |  |  |  |  |  |  |
|  | Delivery Format |  |  |  |  |  |  |
|  | Delivery Setting |  |  |  |  |  |  |
|  | Educator Role |  |  |  |  |  |  |
|  | Theory/Framework Used |  |  |  |  |  |  |
|  | Adverse Events |  |  |  |  |  |  |
| **Outcomes and Findings** |  |  |  |  |  |  |  |
|  | Outcomes Measured (completeness, adherece, knowledge retention, quality of life, psychological outcomes, adherence, caregiver stress, disease burden, etc.) |  |  |  |  |  |  |
|  | Measurement Tools |  |  |  |  |  |  |
|  | Key Findings |  |  |  |  |  |  |
| **Quality of Patient Education (refer to PEMAT)** |  |  |  |  |  |  |  |
|  | Understandability |  |  |  |  |  |  |
|  | Actionability |  |  |  |  |  |  |
|  | Credibility |  |  |  |  |  |  |
|  | Cultural Relevance |  |  |  |  |  |  |
|  | Engagement Level |  |  |  |  |  |  |
|  | Satisfaction |  |  |  |  |  |  |
|  | Feasibility |  |  |  |  |  |  |
| **...** |  |  |  |  |  |  |  |
